# Phytochemical Profile and Evaluation of Antioxidant Activity, Enzyme Inhibition and Cell Viability of Leaves Extracts of Three Tunisian Varieties of *Diospyros kaki* L.

**DOI:** 10.1002/fsn3.70775

**Published:** 2025-08-10

**Authors:** Amna Mannai, Asma Ressaissi, Abderrahmen Merghni, Hassiba Chahdoura, Wissem Mnif, Zarah Alelyani, Mossadok Ben‐Attia, Safia EL‐Bok, Maria Luisa Serralheiro

**Affiliations:** ^1^ Laboratory of Biodiversity, Biotechnologies and Climate Change (LR11/ES09), Faculty of Sciences of Tunis Tunis El‐Manar University Tunis Tunisia; ^2^ BioISI–Instituto de Biosistemas e Ciências Integrativas, Faculdade de Ciências Universidade de Lisboa Lisboa Portugal; ^3^ Laboratory of Antimicrobial Resistance LR99/ES09, Faculty of Medicine of Tunis University of Tunis El Manar Tunis Tunisia; ^4^ Institut National des Technologies et des Sciences du El Kef Université de Jendouba El Kef Tunisie; ^5^ Department of Chemistry, College of Sciences at Bisha University of Bisha Bisha Saudi Arabia; ^6^ Environment Biomonitoring Laboratory (LR01/ES14), Faculty of Sciences of Bizerte University of Carthage Bizerte Tunisia; ^7^ Faculdade de Ciências. Departamento de Química e Bioquímica Universidade de Lisboa Lisboa Portugal

**Keywords:** 3‐hydroxy‐3‐methyl‐glutaryl‐coenzyme A reductase, acetylcholinesterase, antioxidant activity, cytotoxicity, *Diospyros kaki*, flavonoids, metabolites, persimmon, total phenolics

## Abstract

Because of their biological qualities, the leaves of *Diospyros kaki* L., also known as persimmon, have long been used in traditional Chinese medicine for the treatment of ischemic stroke, angina, internal hemorrhage, hypertension, atherosclerosis, and some infectious diseases. It has also been used in cosmetics, as well as in refreshing drink preparations. In the present study, we have compared the aqueous extract effects of the leaves of the three Tunisian varieties of *D. kaki* , namely Triumph, Jiro, and Rojo Brillante, prepared by decoction and then filtered. The obtained filtrates were lyophilized and kept cold until analysis. Phytochemical profile and biological activities were performed on the freeze‐dried fractions of the different varieties of *D. kaki* . Analysis of the results shows that the leaf extracts of the three varieties are rich in total phenols and flavonoids (ranking: Rojo Brillante > Triumph > Jiro) and have a high antioxidant activity (EC_50_ values of 3.8, 8.0 and 12.1 μg/mL respectively for Rojo Brillante, Triumph and Jiro). Using a quadrupole time‐of‐flight interface and high‐resolution tandem liquid chromatography, 29 secondary metabolites of *D. kaki* leaf decoctions were identified in the three varieties, including several polyphenols such as flavonoids, tannins, organic acids, and others. In addition, these extracts were found to inhibit the function of two key enzymes, acetylcholinesterase (IC_50_ values of 58, 96 and 210 μg/mL respectively for Rojo Brillante, Triumph and Jiro) and inhibition percentages of 3‐hydroxy‐3‐methylglutaryl‐CoA reductase were 61.44%, 57.35%, and 46.28% for Jiro, Triumph, and Rojo Brillante, respectively. However, we noted that these different extracts had no cytotoxic effect on human liver or breast cancer cells in culture until 10 μg/mL. The results reveal that the *D. kaki* leaves are a potential matrix for the launch of future nutraceuticals, cosmetics, and pharmaceutical specialties such as drugs to combat Alzheimer's disease or to reduce cholesterol levels, as well as an attractive source of ingredients beneficial to health. Comparative analysis of *D. kaki* leaves showed that the Rojo Brillante variety has a higher content of secondary metabolites than the other two varieties, thus increasing its antioxidant activity and enzyme inhibitory effect, although it is not as cytotoxic as the other two varieties.

## Graphical abstract

Aqueous leaf extracts of three *Diospyros kaki* varieties (Triumph, Jiro, Rojo Brillante) showed significant antioxidant activity, enzyme inhibition (AChE & HMGR), and lacked cytotoxicity. LC–MS–QTOF identified 29 phenolic compounds, with Rojo Brillante being the richest. These bioactivities support potential applications in preventing neurodegenerative and cardiovascular diseases.

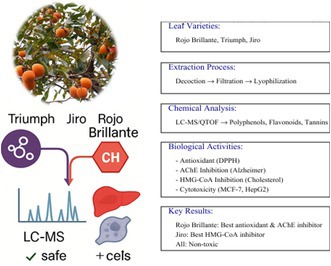

## 1 Introduction

The Chinese persimmon ( *Diospyros kaki* L.), which belongs to the *Ebenaceae* family, has been cultivated in East Asia for centuries. Because of its healing qualities, persimmon leaves have been used extensively in Chinese medicine (Xie et al. 2015; Kurt and Kaya 2020). Persimmon leaves are generally used in infusions as a beverage, and the fruit can be eaten in any way (Guan et al. 2021). Numerous studies have been carried out to extract bioactive substances from the aerial parts of this plant, such as polyphenols, known for their pharmacological benefits (Batiha et al. 2020). Research has shown that persimmon leaf extracts possess a very wide range of therapeutic properties, such as antioxidant, antiacetylcholinesterase, and antidiabetic properties. They have been implicated in the treatment of ischaemic stroke, angina pectoris, internal bleeding, hypertension, atherosclerosis, and certain infectious diseases (Xie et al. 2015; Kurt and Kaya 2020). However, research has largely linked these biological activities to polyphenols (i.e., flavonoids, phenolic acids and tannins). More specifically, the flavonoids that are contained in persimmon leaves, such as quercetin, kaempferol, and catechin, have been shown to have powerful antioxidant properties and a good capacity to control arterial hypertension (Esteban‐Muñoz et al. 2021). In addition, persimmon quercetin has been shown to have anticancer activity, modifying cell cycle progression by suppressing cell proliferation and promoting apoptosis, as well as blocking angiogenesis and the formation of metastases. It has also affected autophagy and has effectively treated prostate and breast cancers (Ding et al. 2017; Tang et al. 2020).

Alzheimer's disease (AD) is an evolutive neurodegenerative pathology characterized mainly by the degeneration of cholinergic neurons. The key enzyme, acetylcholinesterase (AchE: E.C.3.1.17) is a hydrolase enzyme with esterase activity, which hydrolyzes acetylcholine into choline and acetate (Lorena et al. 2022). Treatment of AD includes inhibitors of acetylcholinesterase, butyrylcholinesterase, and amyloid precursor protein beta cleavage enzyme 1 (Zhang et al. 2019). Several studies have also reported that certain plants rich in phenolic compounds, such as kaempferol, exert an inhibitory activity on AChE and appear to have protective effects in the face of Alzheimer's disease (Kim et al. 2020).

Hypercholesterolemia is a metabolic disorder marked by elevated cholesterol levels in the bloodstream. This phenomenon may be ascribed to excessive cholesterol, saturated fats, high caloric content, or hereditary predispositions. Elevated cholesterol levels in the blood (Röhrl and Stangl 2018) can result in metabolic disorders like obesity, diabetes, and perhaps even cancer (Gholamhoseinian et al. 2010). One of the many ways in which the body controls cholesterol homeostasis is by stopping 3‐hydroxy‐3‐methyl‐glutaryl‐coenzyme A (HMG‐CoA) reductase (HMGR), the enzyme responsible for producing endogenous cholesterol from the initial cholesterogenesis reaction (Baskaran et al. 2015). In addition to statins, as HMGR inhibitors, several phenolic compounds are also capable of inserting themselves into the active site of this enzyme and could act to slow down cholesterol biosynthesis (Arantes et al. 2016; Ressaissi et al. 2016; Guedes et al. 2019; Fadel et al. 2020).

Another important biological activity is cellular antioxidant activity, which helps to reduce inflammatory processes and prevent the production of reactive oxygen species (ROS). ROS are responsible for many diseases, including cancer, cardiovascular disorders, and neurodegenerative diseases (Siti et al. 2015; Mileo and Miccadei 2016). Several studies have shown that oxidative stress alters gene expression in cell proliferation and apoptosis. Other studies have also highlighted the role of ROS in tumor growth and dissemination (Mileo and Miccadei 2016). The variance or mismatch between cell production and cell apoptosis (programmed cell death), which indicates an imbalance in the cell cycle, increases the aggressive behavior of tumor cells, including the proliferation and survival of cancer cells, which are thought to be the only important functions of tumor cells (Morana et al. 2022). Restoring the balance between proliferation and apoptosis is very promising, as it involves preventing or blocking cell proliferation and encouraging apoptosis within a cell cycle. This could represent a significant anticancer therapeutic target (Kurgan et al. 2017). Epidemiologists believe that consuming foods rich in antioxidant plants may help to reduce certain cancers (Liu et al. 2009).

The objective of this study was to identify the phenolic acids contained in the leaves of three varieties of *D. kaki* : Triumph, Jiro, and Rojo Brillante and thus to assess their efficacy as antioxidants and inhibitors of enzymatic activities (in the case of AchE and HMGR), as well as to evaluate the cytotoxicity of the substances contained in the leaf extracts of these three Tunisian varieties on cell lines in culture (breast cancer cells and human liver cells).

## 2 Materials and Methods

### 2.1 Chemicals

All the chemicals were of analytical grade. Sigma‐Aldrich (Barcelona, Spain) provided the HMGR assay kit (CS1090) and AChE (149 U/mg solid, 241 U/mg protein). Methanol and ethanol (≥ 99.8%) from Riedel‐de Haën (Charlotte, North Carolina, USA). Thermo‐Fisher Scientific (Waltham, Massachusetts, USA) provided HPLC–grade acetonitrile. We got the trypsin, glutamine, phosphate‐buffered saline (PBS), Dulbecco's modified Eagle medium (DMEM) with 4.5 g/L glucose (high glucose), and fetal bovine serum (FBS) from Lonza in Basel, Switzerland.

### 2.2 Plant Material

Leaves of *Diospyros kaki* L. were gathered from orchards in north‐eastern and north‐western Tunisia: Beja (GPS coordinates: ~38°0′55.094″ N 7°51″ 45.832′ W), Mannouba (36° 48′ 33.581′ N 10° 5′ 10.777′ E), Zhagouen (36° 24′ 32.828′ N 10°8′ 32.341′ E), and Nabeul (36°27′ 18.238′ N 10° 42′ 55.523′ E). The plant varieties were identified by the botanist Mounir Kasri (INAT, University of Carthage). Leaf samples representing the three varieties were submitted to the laboratory for analysis. Each variety was assigned a unique identification number and the corresponding samples were deposited in the laboratory's herbarium. Accordingly, the Triumph variety was assigned the number VF‐01, the Jiro variety the number VF‐02, and the Rojo Brillante variety the number VF‐03. In this study, we used the leaves of three different varieties of *D. kaki* : Triumph, Jiro and Rojo Brillante after cleaning with ice‐cold distilled water to remove surface contaminants, prevent enzymatic activity, minimize mechanical damage, and prevent degradation, we left them to dry in the shade at room temperature for 7 days. On the 8th day, the leaves were ground to a fine powder.

### 2.3 Extraction

Aqueous extracts of the leaves of the three varieties of *D. kaki* were prepared by decoction with 10 g of leaf powder boiled in 300 mL of distilled water for 15 min in a covered container. The decoctions were filtered using Whatman paper, and the resulting filtrates were freeze‐dried with a Heto PowerDry LL3000 freeze‐dryer. The extraction yield ranged from 2.5% to 3%. All preparations were kept cold and used within 24 h.

### 2.4 Secondary Metabolites Analysis

Secondary metabolites of the extracts were analyzed using LC–MS–QTOF, following the methodology outlined by Guedes et al. (2019). The Metlin database and Data Analysis software were used to annotate chemical formulae on the basis of exact mass. Thus, extracts were analyzed in both positive and negative modes; however, only the negative mode was utilized for the dereplication process due to the necessity for precise annotation of molecular characteristics based on mass measurement.

For LC–MS‐QTOF analysis, 0.5 mg of each extract was dissolved in Milli‐Q water. Chromatographic separation was carried out using an Intensity Solo 2 C18 column (100 × 2.1 mm, 1.8 μm; Bruker Daltonics), which was maintained at 35°C. A flow rate of 0.25 mL/min was used, and 5 μL of each sample was injected. Elution was performed using a mobile phase consisting of water with 0.1% formic acid (eluent A) and acetonitrile with 0.1% formic acid (eluent B), according to the following gradient: 95% A/5% B at 0 min; 25% A/75% B at 1.5 min; 100% B at 13.5 min; and re‐equilibration to 95% A/5% B at 21.5 min.

Mass spectrometry was performed using both positive and negative ion modes, with high‐resolution settings in place. We set the ion spray voltage to −3.5 kV and adjusted the endplate offset to 500 V. The nebulizer gas pressure was at 29 psi, while the dry gas flow rate was 4.0 L/min, and the source temperature was maintained at 200°C. For mass calibration, we followed a high‐precision internal method using a mixed solvent solution. Data was collected in full scan mode, ranging from 50 to 1500 *m*/*z* at a frequency of 1 Hz. We also employed automatic MS/MS for ion fragmentation, analyzing each sample in triplicate to ensure consistent results.

### 2.5 Total Phenol and Flavonoid Contents

The total phenolic content of *Diospyros kaki* aqueous extracts was determined using the Folin–Ciocalteu method described by Pisoschi and Pop (2015), with slight modifications. 20 μL of each extract (5 mg/mL) was added to 30 μL of Folin–Ciocalteu reagent and 1350 μL of distilled water. The mixture was gently mixed, and after 3 min, 90 μL of 2% aqueous sodium carbonate (Na_2_CO_3_) solution was added. The reaction mixture was then incubated at 4°C with stirring for 2 h. The absorbance was measured at 760 nm. A standard calibration curve was constructed using gallic acid at concentrations ranging from 10 to 1000 μg/mL under identical experimental conditions. Total phenolic content was expressed as micrograms of gallic acid equivalents per milligram of extract (μg GAE/mg extract). Absorbance values were converted to gallic acid concentrations according to the standard curve.

Total flavonoid content was estimated following the aluminum chloride colorimetric method adapted from Tai et al., as cited in Dzoyem et al. (2015). Briefly, 20 μL of each extract (5 mg/mL) was mixed with 300 μL of 5% sodium nitrite (NaNO₂) and 480 μL of distilled water. After standing at room temperature for 5 min, 30 μL of 10% aluminum chloride (AlCl₃) was added. Exactly 1 min later, 200 μL of 1 M sodium hydroxide (NaOH) was introduced. The mixture was adjusted to a final volume of 1 mL with distilled water, vortexed, and absorbance was measured at 510 nm. A calibration curve was prepared using catechin standards over a range of 10–1000 μg/mL. Flavonoid content was expressed as milligrams of catechin equivalents per gram of dry extract (mg CE/g). All assays were carried out in triplicate.

### 2.6 Antioxidant Activity

The DPPH (1,1‐diphenyl‐2‐picrylhydrazyl) radical scavenging activity of aqueous extracts from the three *D. kaki* varieties was assessed using the method described by Katalinic et al. (2006). For the assay, 10 μL of each extract at different concentrations was mixed with 990 μL of DPPH (0.002% in methanol) and was left to react at room temperature in the dark for 30 min. Absorbance measurements were recorded at 517 nm using a UV–Vis spectrophotometer. The antioxidant activity of different extracts was evaluated by the EC_50_ (determined by a specific time) using the method of Falé et al. (2014). DPPH was dissolved in methanol, and different dilutions were carried out to obtain 0.05, 0.1, 0.5, 1, 2.5, 5.00 mg DPPH solutions that served as a calibration curve.

### 2.7 Acetylcholinesterase and HMGR Inhibition

The acetylcholinesterase (AChE) inhibitory activity of aqueous *Diospyros kaki* (persimmon) extracts was evaluated following the protocol adapted from Falé, Amaral, et al. (2012) and Falé, Ascensão, et al. (2012). Reaction mixtures were prepared by combining 350 μL of Tris–HCl buffer (50 mM, pH 8.0), 10 μL of extract at varying concentrations, and 25 μL of AChE solution (0.26 U/mL in Tris–HCl, pH 7.5), followed by pre‐incubation at 25°C for 15 min. The reaction was initiated via sequential addition of 75 μL of 15 mM acetylthiocholine iodide and 475 μL of 3 mM DTNB reagent. Absorbance was measured at 405 nm, and enzyme activity inhibition was quantified relative to a water‐based negative control. The concentration range of the acetylcholinesterase calibration curve is 0.5, 1, 2.5, and 3 mg/mL, which served as the calibration curve.

For the measurement of HMGR enzymatic activity by HPLC, we followed the instructions of the supplier (Sigma‐Aldrich, CS1090). The technique developed by Mozzicafreddo et al. (2010) was employed to quantify hydrogen nicotinamide adenine dinucleotide phosphate (NADPH), as adapted by Falé, Amaral, et al. (2012) and Falé, Ascensão, et al. (2012). The reaction mixture, which consisted of HMGR (0.4 μM), NADPH (2.68 mM), and HMG‐CoA (1.55 μM) diluted in activity buffer, was incubated at 37°C and sampled at 0, 1, 2, 4, and 6 min (Mozzicafreddo et al. 2010). The reaction was terminated by adding 50% methanol, and the amount of NADPH was quantified by HPLC‐DAD, using a Hitachi Elite LaChrom VWR jewel liquid chromatograph with an L‐2300 column oven and L‐2455 diode array detector (VWR, USA), and with a LiChroCART 250–4 LiChrospher 100 RP‐18 (5 μm) column. Twenty‐five microliters were injected, and the method consisted of a gradient of solution A (100 mM KH_2_PO_4_) and solution B (methanol) as follows: 0 min, 95% A, 5% B; 15 min, 70% A, 30% B; 20 min, 20% A, 80% B; 23 min, 20% A, 80% B, at a flow rate of 0.800 mL/min. The assays were done in triplicate. The level of inhibition in the presence of persimmon leaf extracts was determined by the percentage decrease in activity compared with the absence of inhibitor (100% activity value).

### 2.8 Cytotoxicity

Two cell lines were used for cytotoxicity studies: Hep G2 (ATCC HB‐8065, human hepatocellular carcinoma cell line) and MCF‐7 (ATCC HTB‐22, human mammary gland adenocarcinoma epithelial cell line). Cells were grown at 37°C in a 5% CO_2_ atmosphere in DMEM enriched with 10% FBS, 100 U/mL penicillin, 100 U/mL streptomycin, and 2 mM l‐glutamine. Prior to confluence, cells were passaged every 48–72 h. Cytotoxicity of aqueous *D. kaki* leaf extracts was assessed on the MTT viability assay (Ressaissi et al. 2017). Cells were seeded into 96‐well plates at a density of 1 × 10^4^ cells/well to allow cells to adhere overnight. They were incubated for 24 h in a 5% CO_2_ atmosphere (at 37°C). After the incubation period, 10 μL of MTT solution (5 mg/mL; diluted in saline) was added to each well, and the plates were incubated for 4 h to allow formazan crystal formation. 100 μL of 96% ethanol was added to dissolve the formazan crystals, and the absorbance was read at 595 nm using a Bio‐Rad iMark microplate reader. The cells were exposed to different concentrations (0.05–1 mg/mL) of plant extracts for 48 h. All studies were performed in three replicate wells.

### 2.9 Statistical Analysis

Data were presented as the mean ± standard deviation of at least three separate measurements. A Tukey multi‐range test was performed after a one‐way analysis of variance (ANOVA) was applied to the experimental data. Origin Pro 2020b was used to perform the analysis. A P value less than or equal to 0.05 was considered statistically significant.

## 3 Results

### 3.1 Total Polyphenol Content of *Diospyros kaki* Leaves

The total phenolic concentrations, in mg GAE/mg extract, in the three types of extracts were 359.77 ± 6.42 (Triumph), 325.19 ± 7.48 (Jiro), and 490.91 ± 5.19 (Rojo Brillante) (Table 1). The varieties Jiro, Rojo Brillante, and Triumph had flavonoid contents of 126.1 ± 6.3, 223.1 ± 4.4, and 293.2 ± 4.8 μg EC/mg extract, respectively. Rojo Brillante exhibited the maximum flavonoid concentration, with 293.25 ± 4.8 μg CE/mg extract, succeeded by Triumph. In contrast to the other two kinds, Jiro exhibited the lowest flavonoid content, with 126.06 ± 6.3 μg CE/mg extract (Table 1).

**TABLE 1:** Contents of total flavonoids and total phenols in extracts from persimmon leaves.

Variety	Total phenols (μg GAE/mg extract)	Total flavonoids (μg CE/mg extract)
Triumph	359.77 ± 6.42^a^	223.05 ± 4.498^a^
Jiro	325.19 ± 7.48^b^	126.063 ± 6.377^b^
Rojo Brillante	490.91 ± 5.19^c^	293.252 ± 4.894^c^

*Note:* ^a,b,c^Different letters mean significantly different at *p* ≤ 0.05.

### 3.2 Phenolic Compounds Identification by LC–MS/QTOF

**FIGURE 1:**
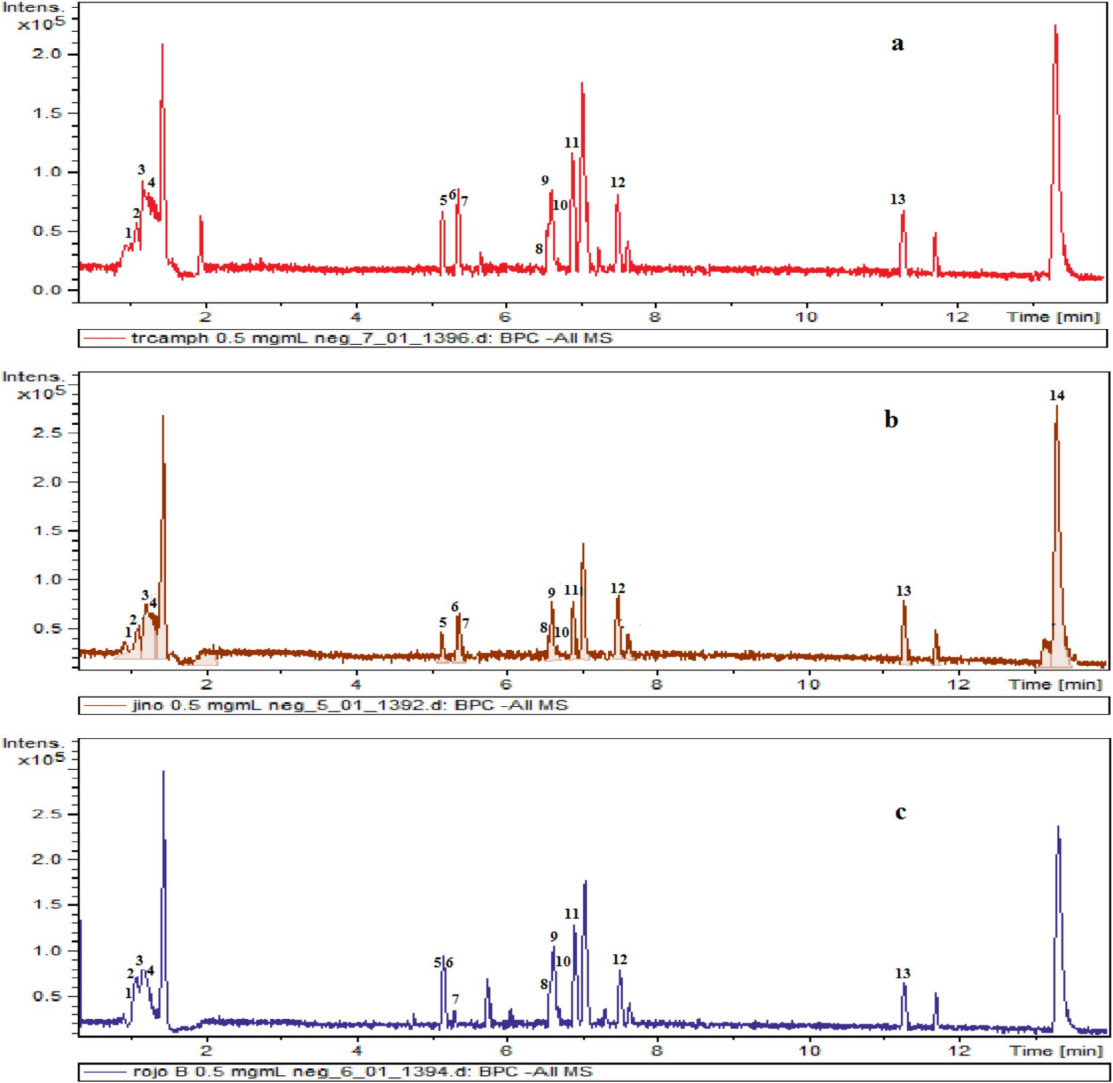
Chromatogram of persimmons processed in the negative ion mode using LC–MS‐QTOF. These three lemons are called Rojo Brillante (c), Jiro (b), and Triumph (a).

The phenolic fraction of three varieties of persimmon leaves was analyzed using LC–MS–QTOF. Figure 1 displays the chromatograms of the three persimmon varieties.

In total, 28 polyphenols, one benzoic acid, 15 flavonols, seven flavones, three isoflavonoids, one flavanone, and one methoxyphenol were identified (Table 2). The results of this qualitative screening of persimmon extract polyphenols are summarized in Table 2, which reveals variations in phenolic components between the three varieties. Parent ion fragmentation [M–H] confirmed the differences in probable metabolites. Our research suggests that persimmon leaves possess a complex phenolic composition, potentially enhancing their advantageous effects in traditional ethnomedicine.

**TABLE 2:** Identified molecules in persimmon leaf extracts by LC–MS–QTOF in negative ion mode.

Peak	Rt (min)	Compound (subclass)	Molecular formula	Accurate [M–H]^−^ *m/z* (Δppm)	Fragments *m*/*z* (% intensities)
1	1.1	8‐Hydroxydaidzein (isoflavones)	C_15_H_10_O_5_	269.0434 (−3.7)	88.9824 (100)
1	1.1	6,8‐Di‐C‐methylkaempferol 3,7‐dimethyl ether (flavonols)	C_15_H_18_O_9_	341.1005 (−7.62)	165.0355 (100)
1	1.1	7‐Hydroxy‐3′,4′, 5, 6,8‐pentamethoxyflavone (flavones)	C_20_H_20_O_8_	387.1038 (−6.4)	89.0192 (100); 119.0536 (64) 149.0397 (24); 179.0503 (42)
1	1.1	Benzoyl benzoate (benzoic acid)	C_14_H_10_O_3_	225.0547 (0.44)	59.0091 (100); 89.0192 (30)
2	1.2	5‐Hydroxy‐3,7,8,2′,4′‐pentamethoxyflavone (flavones)	C_20_H_20_O_8_	387.1047 (0.51)	59.0085 (100); 89.0192 (84) 341.1023 (32)
2	1.2	5,7,8‐Trihydroxyflavone 7‐galactoside (flavones)	C_21_H_20_O_10_	431.0921 (−11.8)	89.9844 (100)
2	1.2	7,2′‐Dihydroxy‐3′,4′‐dimethoxyisoflavone 7‐O‐glucoside (flavones)	C_23_H_24_O_11_	475.1173 (−12.8)	133.0090 (100); 114.9983 (40); 135.0244 (38)
3	1.3	5,2′,5′‐Trihydroxy‐3, 6,7, 4′‐tetramethoxyflavone 5′‐glucoside (flavones)	C_25_H_28_O_14_	551.1310 (−15.4)	95.9487 (100); 78.9517 (60)
3	1.3	Kaempferol 3‐rhamnoside (flavonols)	C_21_H_20_O_10_	431.0924 (−13.91)	88.9827 (100)
4	1.5	Quercetagetin 3, 5, 6, 7,3′‐pentamethyl ether (flavonols)	C_20_H_20_O_8_	387.1085 (2.0)	89.0190 (100); 341.0999 (58) 179.0502 (41); 113.0196 (20)
5	5.1	(+)‐Catechin (flavonols)	C_15_H_14_O_6_	289.0635 (−24.5)	109.0242 (100); 123.0400 (80) 203.0649 (57); 151.0353 (40)
5	5.1	Kaempferol 3‐O‐xylosyl‐glucoside (flavonols)	C_26_H_28_O_15_	579.1339 (0.86)	289.0640 (100); 245.0750 (11)
6	5.2	3,5,8‐Trimethoxy‐6,7:3′,4′‐bis(methylenedioxy)flavones (flavones)	C_20_H_16_O_9_	399.0827 (29.3)	191.0287 (100); 152.0162 (13)
7	5.4	5,3′,5′‐Trihydroxy‐3, 6, 7, 8,4′‐penta‐methoxyflavone (flavones)	C_20_H_20_O_10_	419.1085 (26.9)	89.0190 (100); 141.0141 (82); 71.0084 (74); 119.0301 (57)
7	5.4	Daidzein 6″‐Oacetate (isoflavonoids)	C_23_H_22_O_10_	457.1240 (24.3)	119.0450 (100) 163.043 (83)
8	6.6	Quercetagetin 3′‐methylether 6‐glucoside (flavonols)	C_22_H_22_O_13_	493.0869 (−21.7)	315.0062 (100); 287.0124 (84) 330.0273 (50); 301.0257 (43)
8	6.6	Quercetin 7‐(6″‐galloylglucoside) (flavonols)	C_28_H_24_O_16_	615.0812 (−27.3)	301.0282 (100); 150.9985 (17)
8	6.6	6‐Hydroxykaempferol 3‐glucoside (flavonols)	C_21_H_20_O_12_	463.0758 (−24.4)	300.0212 (100);271.0176 (20) 463.0765 (19);243.0236 (13)
8	6.6	Oolonghomobisflavan B (flavonols)	C_45_H_36_O_22_	927.1555 (−6.4)	463.0744 (100); 300.0196 (87)
8	6.6	Quercetin 3galacturonide (flavonols)	C_21_H_18_O_13_	477.0540 (−25.7)	301.0266 (100); 150.9981 (15)
9	6.8	Kaempferol 7‐O‐glucoside (flavonols)	C_21_H_20_O_11_	447.0815 (−22.2)	284.0261 (100); 227.0288 (71)
10	6.9	Kaempferol 7‐(6″‐galloylglucoside) (flavonols)	C_28_H_24_O_15_	599.0872 (−26.5)	285.0329 (100); 313.0478 (33)
10	6.9	Quercetin 3‐O‐xyloside (flavonols)	C_20_H_18_O_11_	433.0664 (−23.3)	300.0197 (100); 271.0166 (19)
11	7	Kaempferol 5‐glucuronide (flavonols)	C_21_H_18_O_12_	461.0596 (25.5)	285.0335 (100)
12	7.5	5,6,7,2′,3′,4′,5′‐Heptamethoxyflavanone (flavanones)	C_22_H_26_O_9_	433.1505 (2.7)	91.0348 (100); 249.0602 (98)
12	7.5	Daidzein 7‐O‐glucoside‐4′‐O‐apioside (isoflavonoids)	C_26_H_28_O_13_	547.1393 (−12.4)	249.0605 (100); 363.0864 (84) 160.0095 (51)

### 3.3 Antioxidant Activity

The results of the DPPH radical‐scavenging experiments performed on each extract are shown in Table 3. The Jiro extracts (EC_50_ = 12.05 ± 0.11 μg/mL) and Triumph (EC_50_ = 8.01 ± 0.35 μg/mL) demonstrated lower antioxidant activity than the Rojo Brillante extract (EC_50_ = 3.79 ± 0.19 μg/mL).

**TABLE 3:** Antioxidant activity and AChE inhibitory activity of the three varieties of persimmon leaves.

Variety	DPPH EC_50_ (μg/mL)	AChE IC_50_ (μg/mL)
Triumph	8.01 ± 0.35^a^	95.98 ± 16.3^a^
Jiro	12.05 ± 0.11^b^	210.21 ± 28.9^b^
Rojo Brillante	3.79 ± 0.19^c^	58.35 ± 0.69^c^
BHT	3.39 ± 0.04	
Galantamine		0.24 ± 0.03

*Note:* ^a,b,c^Different letters mean significantly different at *p* ≤ 0.05.

### 3.4 Acetylcholinesterase Inhibition

The results of the present study showed that AChE inhibitory activity increased in a concentration‐dependent manner with the extracts of each of the three *D. kaki* varieties (Table 3). In comparison to galantamine (positive control: IC_50_ = 8.2 ± 2.73 μg/mL), the extract from the Jiro variety exhibited the lowest activity, with an IC_50_ value of 210.21 ± 28.9 μg/mL. Conversely, the Rojo Brillante variety demonstrated the highest inhibition, with an IC_50_ value of 58.35 ± 0.69 μg/mL, while the Triumph extract exhibited an intermediateIC_50_ value of 95.98 ± 1.63. Statistical analysis revealed a significant differences (*p* < 0.05 by Tukey's test) between all pairs of leaf extracts from the three *D. kaki* varieties, that is, Jiro, Triumph, and Rojo.

### 3.5 HMG‐CoA Reductase Inhibition

**FIGURE 2:**
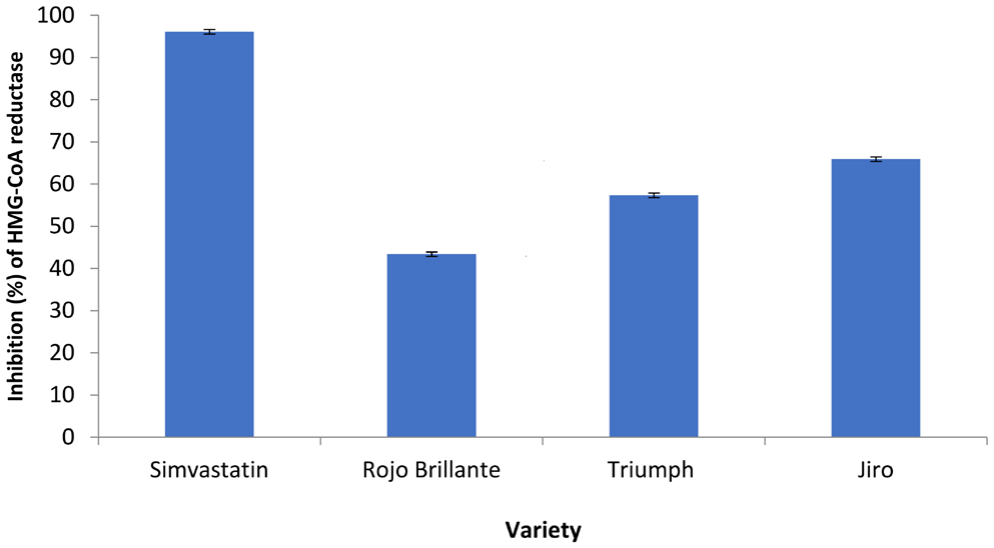
Inhibition (%) of HMG‐CoA reductase for 100 μg/mL of persimmon leaf extracts from three varieties (Triumph, Jiro, and Rojo Brillante) of *Diospyros kaki* .

Reducing cholesterol biosynthesis is probably the most effective way of lowering plasma cholesterol levels. Aqueous extracts of *D. kaki* leaves, at a concentration of 100 μg/mL, showed good HMG‐CoA reductase inhibitory activity (Figure 2). The extracts had, an enzyme inhibition percentage of 61.44%, 57.35%, and 46.28% respectively for Jiro, Triumph, and Rojo Brillante, varieties. The results obtained revealed the ability of these extracts to have a hypocholesterolemic effect by inhibiting this rate‐limiting enzyme in cholesterol biosynthesis.

### 3.6 Cytotoxicity

**FIGURE 3:**
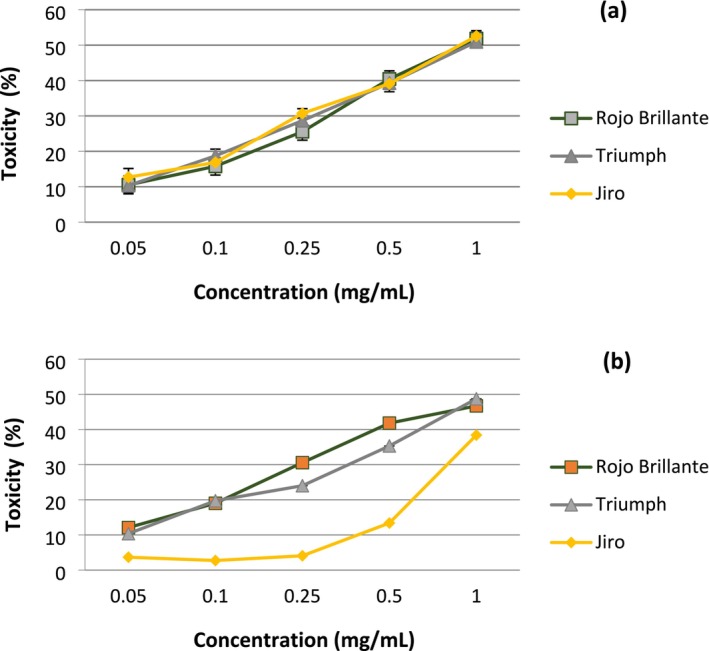
Effects of leaf extracts of the three varieties of *Diospyros kaki* on the cytotoxicity of two cell lines: (a) MCF‐7 and (b) HepG2. According to the U.S. National Cancer Institute, extracts are considered potentially cytotoxic if their IC_50_ value is below 20 μg/mL; values above this threshold are regarded as noncytotoxic.

The cytotoxicity of aqueous persimmon leaf extract was tested with five increasing concentrations, ranging from 0.05 to 1 mg/mL, on human HepG2 and MCF‐7 cell lines (Figure 3). Extract concentrations killing 50% of cells (IC_50_) were determined from dose–response curves. For the HepG2 cell line, IC_50_ values ranged from 1 to 1.47 mg/L; 1.47 was calculated by extrapolation of the curve. IC_50_ values were 0.96, 0.98, and 1.47 mg/mL for Rojo Brillante, Triumph, and Jiro, respectively. For the MCF‐7 cell line, calculated IC50 values for Rojo Brillante, Triumph, and Jiro were 0.84, 0.85, and 0.94 mg/mL, respectively.

The results indicated that the extracts examined exhibited no cytotoxicity on HepG2 and MCF‐7 cells, since the IC_50_ values exceeded those established by the US National Cancer Institute as being cytotoxic. According to the standards of this institute, an IC_50_ value greater than 20 μg/mL does not indicate a possible cytotoxic action of the extracts tested.

## 4 Discussion

Phenolic compounds, the most important antioxidants, are present in many plant species. The study of the antioxidant activity of plant‐derived nutraceuticals is receiving particular attention. Their biologically active secondary metabolites are used in the research and development of plant protection products such as biopreservatives, pharmaceuticals, and biopesticides. The antioxidant activity of plants has been extensively studied using volatile and nonvolatile extracts, obtained by various extraction solvents from different parts of the plants studied, such as *Terfezia claveryi*, *Rubia tinctorum* L., *Hypericum heterophyllum*, and *Allium vineale* (Hadjira et al. 2024; Houari et al. 2024; Yaman et al. 2024; Demirtas et al. 2013), which exhibit widely documented antioxidant activity. Thus, the presence of phenolic compounds in persimmon could enhance the protective properties of the extracts, which requires the evaluation of the total phenolic concentration in the extracts of three Tunisian varieties of *D. kaki* (persimmon). In addition, the bioactive substances contained in persimmon leaves are interesting in that they have been described as endowed with beneficial health effects by Anand and Singh (2013).

In fact, analysis of leaf extracts from three *D. kaki* varieties revealed high levels in phenolic compounds with a total phenolic content of 490.9 ± 5 μg GAE/mg for the extract of the Rojo Brillante variety, which had the highest content of the three varieties analyzed. Conversely, the Jiro variety had the lowest content (325.2 ± μg GAE/mg extract). The polyphenol content of *D. kaki* varieties grown in Tunisia is the highest in comparison with literature results (Chang et al. 2019; Esteban‐Muñoz et al. 2021). In particular, Esteban‐Muñoz et al. (2021) found that the Triumph variety, a nonastringent persimmon, and Rojo Brillante, an astringent persimmon, both had average total phenolic concentrations ranging from 84.57 to 380.79 μg GAE/g, respectively.

The Jiro variety had the lowest average flavonoid content, at 126.06 ± 6.38 μg catechin equivalent (CE)/mg extract, while the variety Rojo Brillante had the highest average concentration, which is 293.25 ± 4.89 μg CE/mg extract. On the other hand, Sun et al. (2011) measured the average flavonoid content in persimmon ( *D. kaki L. folium* ) leaf extracts, which was around 192‐μg EC/g extract, lower than that of our Tunisian varieties. In addition, the average contents of our three varieties are significantly higher than those reported by Chang et al. (2019) from persimmon leaves of the eight popular persimmon varieties in Taiwan, between September and November 2017.

In general, the leaves of the persimmon tree contain a wealth of bioactive compounds, which have the potential to exert a variety of positive impacts on one's health (Xie et al. 2015). Twenty‐eight polyphenols in total were identified in our study compared with thirty polyphenols identified by Chang et al. 2019 in the eight popular Taiwanese persimmon varieties. Moreover, in the two studies on Tunisian and Taiwanese varieties, the following polyphenols were essentially found in *D. kaki* leaves: flavonoids and phenolic acids were among the most widely responded polyphenols in *D. kaki* leaves. In the case of the three Tunisian varieties, flavonoids are divided into four categories: flavonols, flavones, isoflavones, and flavanones. Numerous phenolic components, such as tannins, procyanidins, flavonoids, tyrosols, and derivatives of hydroxybenzoic and hydroxycinnamic acids, have been found in the Triumph and Rojo Brillante varieties (Esteban‐Muñoz et al. 2021). Thus, the historical use of persimmon leaves in traditional medicine could be closely linked to their complex phenolic composition, especially rich in different forms of flavonoids, which are considered to be important biologically active agents in a number of diseases. Furthermore, phenolic compounds are one of the antioxidant defense mechanisms involved in environmental stress, which itself generates a high production of free radicals and other oxidizing species. Antioxidant activity appears to be linked to both the content and chemical structure of phenolic compounds (Oktay et al. 2003). Therefore, many disorders can be caused by increased oxidative stress (Siti et al. 2015). Cellular antioxidants have been proposed as a means of preventing the formation of excessive amounts of reactive oxygen species (Heras et al. 2016). However, earlier work on radical scavenging activity and structure–activity relationships has revealed that the position of OH groups in phenolic compounds may be a more important determinant of antioxidant activity than the concentration of these compounds. Studies also reveal that the free forms of glycosylated polyphenols, known as aglycones, have a greater antioxidant capacity and a lower hydrogen release potential than the free forms Tsai et al. (2007). After examining the total amount of phenolic compounds, it was logical to examine and assess the antioxidant activity of persimmon leaf extracts, which is useful for determining the antioxidant potential of the leaves of our three persimmon varieties. This could enable us to assess and standardize the quality of plant raw materials. Our results show that aqueous extracts from the leaves of these three varieties have a higher percentage of DPPH inhibition (i.e., lower IC_50s_) than those of the extracts prepared from the fruits of these three persimmon varieties, suggesting that these leaves have a good antioxidant capacity compared with their fruits (personal communication).

According to our results, the antioxidant activity of leaf extracts from the three Tunisian persimmon varieties does not deviate too much from our positive control, which is butylated hydroxytoluene (BHT) whose EC_50_ value is 3.39 mg/mL ± 0.04 μg/mL. The latter confirms its role as a powerful reference antioxidant, thanks to the low concentration required to scavenge 50% of DPPH radicals. Rojo Brillante extract shows a very similar value to BHT, suggesting comparable antioxidant efficacy. In contrast, Triumph and Jiro extracts have higher EC_50_ values, indicating less capacity to neutralize free radicals in this test. This comparison shows that among these extracts, Rojo Brillante is the most promising in terms of antioxidant activity, followed by Triumph, then Jiro, which remains the least active.

Moreover, plants are known to contain metabolites that are active, often only slightly toxic at the thepapeutic doses used in the treatment of various diseases. Indeed, phenolic compounds can reach the active site and inhibit AChE, as has been demonstrated in numerous studies (Falé, Amaral, et al. 2012; Falé, Ascensão, et al. 2012; Guedes et al. 2019). In another study, aqueous extract of the fruit of *Diospyros lotus* L. showed weaker anti‐AChE activity than that described with leaves (Faiz and Baltas 2016).

In contrast, the ethanolic extract of *D. lotus* L. leaves showed slightly less inhibition than the fruit (Katalinic et al. 2006). Although our three leaf extracts had lower affinities than those described by Kim et al. (2020). Nevertheless, leaf extracts of our three varieties studied, as well as those of *D. lotus* cited above, present a less good affinity than our positive control, galantamine, which is a highly potent AchE inhibitor (IC_50_ is 0.23 μg/mL), which explains its use as a drug in Alzheimer's disease‐related cognitive disorders. In comparison, Triumph, Rojo Brillante, and Jiro extracts have much higher IC_50_ values, indicating that they require much higher concentrations to inhibit 50% of this enzyme activity. Of these extracts, Rojo Brillante is the most active, followed by Triumph, then Jiro, which is the least effective.

In a previous study on HepG2 cells using an ethanolic extract of persimmon fruit, the authors noted an inhibition of HMG‐CoA reductase and a decrease in cholesterol levels in HepG2 cells (Kim et al. 2020). The present study shows that persimmon leaf extracts possess anti‐HMG‐CoA reductase activity. This revealed that *D. kaki* leaf extracts can reduce cholesterol but less effectively than simvastatin, whose IC50 described by Williams et al. (2004) is of the order of 0.20 μg/mL. In addition, phenols such as hydroxycinnamic acid derivatives have been shown to have a direct effect on the HepG2 cell line comparable to that of pravastatin, a drug commonly administered to suppress the HMG‐CoA reductase enzyme (Mozzicafreddo et al. 2010).

When we examined the cytotoxicity of the aqueous extracts of the three types of Tunisian varieties, we found that none of them showed toxic effects on HepG2 or MCF‐7 cells, since their IC_50_ values far exceeded the upper limit authorized by the US National Cancer Institute, which considers that extracts are classified as potentially toxic if their IC_50_ value is less than 20 μg/mL. Our study is in line with that carried out on HepG2 cells (Kim et al. 2020), which examined the cytotoxic effects of an ethanolic extract of astringent persimmon, and the results showed no effect at doses of up to 100 μg/mL.

## 5 Conclusions

This study analyzed the bioactive components of the leaf decoction from three Tunisian varieties of *D. kaki* using LC–MS/MS, identifying a total of 28 distinct phenolic compounds. The extracts demonstrated notable antioxidant activity and definite inhibitory effects on HMGR and AChE. Polyphenols, and flavonoids in particular, are the main source of these biological activities, and this may support their potential application as an alternative treatment. Additional research is required to elucidate the mechanism underlying their antilipid activity, in particular, to evaluate the effects of these extracts on hypercholesterolemia. This information may be beneficial in the formulation of novel pharmaceuticals for the treatment of conditions such as hyperlipidemia, atherosclerosis, and Alzheimer's disease.

## Author Contributions

**Amna Mannai:** conceptualization (equal), data curation (equal), methodology (equal), resources (equal), software (equal), writing – original draft (equal). **Asma Ressaissi:** formal analysis (equal), methodology (equal), software (equal), writing – original draft (equal). **Abderrahmen Merghni:** formal analysis (equal), investigation (equal), methodology (equal), software (equal). **Hassiba Chahdoura:** methodology (equal), software (equal), validation (equal). **Wissem Mnif:** funding acquisition (equal), project administration (equal), resources (equal), supervision (equal), validation (equal), writing – review and editing (equal). **Zarah Alelyani:** funding acquisition (equal), investigation (equal), writing – review and editing (equal). **Mossadok Ben‐Attia:** supervision (equal), visualization (equal), writing – review and editing (equal). **Safia EL‐Bok:** conceptualization (equal), formal analysis (equal), methodology (equal), project administration (equal), resources (equal), supervision (equal), writing – original draft (equal). **Maria Luisa Serralheiro:** formal analysis (equal), methodology (equal), software (equal), supervision (equal), writing – review and editing (equal).

## Conflicts of Interest

The authors declare no conflicts of interest.

## Data Availability

The results presented in this study are accessible on request from the corresponding authors.
